# Strengthening surgical healthcare research capacity in sub-Saharan Africa: impact of a research training programme in Nigeria

**DOI:** 10.3389/fmed.2024.1429168

**Published:** 2024-08-09

**Authors:** Emmanuel A. Ameh, Justina O. Seyi-Olajide, Nkeiruka Ameh, Afieharo Michael, Mohammed AS Abdullahi, Oti Nimi Aria, Nkeiruka Obi, Isaac Chukwu

**Affiliations:** ^1^Department of Surgery, National Hospital, Abuja, Nigeria; ^2^Department of Surgery, Lagos University Teaching Hospital, Lagos, Nigeria; ^3^Department of Obstetrics and Gynaecology, Ahmadu Bello University Teaching Hospital, Zaria, Nigeria; ^4^Department of Surgery, University College Hospital, Ibadan, Nigeria; ^5^Department of Maxillofacial Surgery, University of Maiduguri Teaching Hospital, Maiduguri, Nigeria; ^6^Department of Surgery, River State University Teaching Hospital, Port Harcourt, Nigeria; ^7^Smile Train Africa, Lagos, Nigeria; ^8^Department of Surgery, Federal Medical Centre, Umuahia, Abia, Nigeria

**Keywords:** research capacity, low-resource setting, structured training, impact, surgical plan

## Abstract

**Background:**

Limited research capacity has contributed to the lack of high-quality research from low-and middle-income countries. This is compounded by limited research training opportunities. Research capacity scale-up training was deployed as part of the implementation of the National Surgical, Obstetrics, Anaesthesia, and Nursing Plan for Nigeria. We report the impact of this locally contextualized efforts to scale up research capacity in sub-Saharan Africa.

**Methods:**

This is an evaluation of the training of 65 participants in research, grant writing and manuscript writing and publication. Pre- and post-training surveys using a 5-point Likert scale and open-ended questions were administered to evaluate the impact of the programme.

**Results:**

There were 39 (60%) males and 26 (40%) females aged 26–62 years (median 42 years). Thirty-nine (60%) participants had previous training in research, but only 12 (18.5%) had previously received grant writing training, and 17 (26.2%) had previously received manuscript writing and publishing training. Following training, 45 (70.3%) participants agreed that the training was relevant. The research, grant writing and manuscript writing, and publication components of the training were rated high by the participants (45–59, 70.3–92.2%). However, 41.2% felt that there was not enough time, and 32.4% felt that the training was too comprehensive. Nearly all the participants agreed that the training had improved their skills in research, grant writing and manuscript writing and publication, and more than two-thirds subsequently engaged in informal mentoring of others. Overall, participants achieved success in designing their own research projects and publishing manuscripts and grants. Three (4.6%) of the participants had gone on to become faculty for the research training programme. The three top barriers encountered following training were time constraints (67.3%), lack of funding (36.5%) and not being able to find research collaborators (25%).

**Conclusion:**

Outcome of this training programme is encouraging and highlights the feasibility and potential impact of deploying such programmes in low and middle income countries (LMICs). Despite the positive outcomes, barriers including time constraints, funding limitations, and difficulties in finding research collaborators remain to be addressed. Such training programmes need to be supported to strengthen the research capacity in this and similar settings.

## Introduction

It is estimated that 30% of the global burden of disease is surgical (1). The contribution to this burden from low-and middle-income countries is at best estimated due to poor health information systems and research capacity (2). Research capacity is critical to development but has lagged in low-and middle-income countries compared to high-income countries, with sub-Saharan Africa contributing just 1.1% of global researches in 2013 (3).

Sub-Saharan Africa is currently experiencing significant challenges in surgical healthcare due to several factors, including limited resources, inadequate infrastructure, and a shortage of skilled personnel. Amidst these challenges, building research capacity becomes crucial to drive contextualized solutions and improve patient outcomes. Although progress is being made to address the burden of surgical diseases in the region, there remains a critical gap in research capacity (4). This deficit not only hampers the development and implementation of evidence-based practices but also limits the region’s ability to advocate for policy changes and secure funding for essential surgical services.

In low resource settings, research output is limited both in volume and quality. Much of this is due to limited training in research, poor research infrastructure, and very importantly, inadequate investments by countries in research. One report has noted that despite Africa making up 15% of the world’s population, it accounted for only 1.1% of global investments in research. In addition, sub-Saharan Africa invests 0.44% of GDP in research and development compared to 2.28–3.32% in North America and the European Union (5).

Nigeria’s Federal Ministry of Health prioritized scaling up research capacity as one of the key components of her National Surgical, Obstetrics, Anaesthesia and Nursing Plan (NSOANP) (6). The implementation of this plan has been made feasible through collaboration with nongovernmental organizations (7). North–south partnerships have hitherto contributed to improved research capacity in low-and middle-income countries (8–11). These efforts are rarely focused on the surgical healthcare workforce. In addition, the cost of such research capacity programmes is often not sustainable (11–13). For sustainability, the focus should now be more directed to encouraging locally led training programmes that have the potential to reach more participants at less cost. Nigeria’s NSOANP, therefore, deployed research training for the surgical healthcare workforce to teach the fundamentals of high-quality research, writing and publishing as well as research grants writing. This is a report on the outcome of this training and is intended to highlight the feasibility and impact of locally led and contextualized efforts to scale up research capacity in sub-Saharan Africa.

## Materials and methods

This was a cross-sectional study of 65 participants who participated in research training in Nigeria from August 2021–April 2023. The training was funded by Smile Train Incorporated, New York, a cleft lip and palate focused organisation.

### Participant selection

Participants were from surgical healthcare specialties and specialties involved in comprehensive cleft care in the 6 geopolitical zones of Nigeria. Invitations were sent to the cleft lip and palate team leads at institutions involved in comprehensive cleft care to nominate potential participants for training. In addition, NSOANP team lead asked institutions in areas without a cleft lip and palate team to nominate potential participants from surgical healthcare specialties. Nominated participants were requested to provide a statement on their interest in research and the training, if they had unanalysed research data, and at least two research ideas they would want to work on during the training. Participants who were able to provide this information were then selected for training, while ensuring representation across the 6 geopolitical zones.

### Training programme

The 65 participants were in 6 different groups (10–12) and underwent in-person, intensive five-day interactive and hands-on training:

#### Training programme

The training programme consisted of a full 5-day schedule focusing on research design and conduct, grants writing and grants management, statistics and data analysis, as well as manuscript writing and publishing. The detailed schedule is provided in the attached Supplementary material S1. There was no pre-learning activity before the training. All the 6 groups had the same training schedule. However, based on feedback from earlier groups, later groups were given more examples and templates for Gant chart and grant budgeting. The first training was deployed in 2021.

#### Training faculty

The training faculty consisted of experienced and highly published biomedical researchers and an experienced biomedical statistician. The faculty all had previous experience in research mentoring. There were 5–6 faculty at each training session. The training faculty was composed of only local research experts in surgery and obstetrics and gynaecology, as well as trained biomedical statistician.

### Training evaluation

Pre- and post-training evaluations were completed by the participants immediately before and after the training using a survey format. In addition, a follow-up survey was deployed 2 years after the first training session to evaluate the short-term impact of the training programme (3 months for the last group of participants). The survey consisted of questions structured on a five-point Likert scale as well as open-ended questions to evaluate participants’ knowledge, experience and perceived impact of the training. The surveys were deployed using the Survey Monkey^®^ platform. The pre- and post-training surveys are presented in Supplementary material S2.

All participants who took part in the training were invited to participate in the surveys. Overall, 65 participants completed the pre-training evaluation, 64 completed the post-training evaluation and 52 completed the follow-up evaluation.

### Data analysis

The data were analysed directly from the Survey Monkey^®^ platform using the Statistical Package for the Social Sciences version 20. The 5-point Likert scale (strongly agree, agree, neutral, disagree, strongly disagree; and, very confident, confident, neither confident nor unconfident, unconfident, very unconfident) was compressed to a 3-point Likert scale (agree, neutral, disagree; and, confident, neutral, not confident) for the purpose of analysis. Descriptive data are expressed as percentages, medians, interquartile ranges, and qualitative data by creating themes from the open-ended responses.

## Results

### Demographics

There were 39 (60%) males and 26 (40%) females aged 26–62 years (median 42 years). Sixty-five participants were trained. The participants were from 11 different surgical healthcare and cleft care specialties (Table 1).

**Table 1:** Demographics of 65 research training participants.

Demographic	No. (%)
***Sex***	
Male	39 (60)
Female	26 (40)
Total	65 (100)
***Age in years***	
<31	1 (1.5)
31–40	27 (41.5)
41–50	26 (40)
51–60	9 (113.8)
>60	2 (3.1)
Total	65 (100)
***Specialties***	
Plastic Surgery	13 (20)
Oral and Maxillofacial surgery	12 (18.5)
Anaesthesiology	8 (12.3)
Nursing	7 (10.8)
Orthodontics	5 (7.7)
Obstetrics and Gynaecology	5 (7.7)
Paediatric Surgery	5 (7.7)
Paediatrics	3 (4.6)
Speech Therapy	3 (4.6)
Dentist (paedodontics)	2 (3.1)
Otorhinolaryngology	1 (1.5)
Nutrition	1 (1.5)
Total	65 (100)

### Pre-training evaluation

Thirty-nine (60%) participants had previous training in research, but only 12 (18.5%) had previously received grant writing training, and 17 (26.2%) had previously received manuscript writing and publishing training. In addition, 44 (67.7%) participants had presented a paper at a conference, 33 (50.8%) had a publication as a lead author, and three (4.6%) had received a research grant.

### Post-training evaluation

Following training, 45 (70.3%) participants agreed that the training was relevant, 46 (71.9%) agreed that it was comprehensive, and 47 (73.4%) agreed that it was easy to understand. The individual components of the training were also highly rated by the participants (45–59, 70.3–92.2%). Similarly, 53 (82.8%) participants agreed that the handouts for the training were useful, and 59 (92.2%) agreed that the faculty were knowledgeable and responsive to the participants’ training needs. Eleven (17.2%) participants disagreed that the breaks given during the training were sufficient (Table 2).

**Table 2:** Post-training participants’ feedback on the research training.

Questions	Responses		
	Agree *n* (%)	Disagree *n* (%)	Neutral *n* (%)
**Training content as a whole was?**			
Relevant	45 (70.3)	3 (4.7)	16 (25.0)
Comprehensive	46 (71.9)	2 (3.1)	16 (25.0)
Easy to understand	47 (73.4)	1 (1.6)	16 (25.0)
**The training content on Fundamentals of Research was?**			
Relevant	59 (92.2)	0 (0.0)	5 (7.8)
Comprehensive	58 (90.6)	0 (0.0)	6 (9.4)
Easy to understand	58 (90.6)	1 (1.6)	5 (7.8)
**The training content on Writing for Publication and Grants Writing was?**			
Relevant	47 (73.4)	1 (1.6)	16 (25.0)
Comprehensive	48 (75.0)	0 (0.0)	16 (25.0)
Easy to understand	45 (70.3)	2 (3.1)	17 (26.6)
**The training Handouts?**			
Supported presentation materials	58 (90.6)	1 (1.6)	5 (7.8)
Provided useful additional information	53 (82.8)	5 (7.8)	6 (9.4)
Were clear and well organized	49 (76.6)	10 (15.6)	5 (7.8)
**The training was?**			
Well-paced	56 (87.5)	3 (4.7)	5 (7.8)
A good mix between listening and activities	56 (87.5)	3 (4.7)	5 (7.8)
**Breaks were sufficient**	48 (75.0)	11 (17.2)	5 (7.8)
**Facilitators were?**			
Knowledgeable	59 (92.2)	0 (0.0)	5 (7.8)
Well prepared	59 (92.2)	0 (0.0)	5 (7.8)
Responsive to participant questions	59 (92.2)	0 (0.0)	5 (7.8)
**The activities were useful learning experiences**	48 (75.0)	0 (0.0)	16 (25.0)

The thematic responses on what the participants liked best about the training showed that the faculty and learning structure had the highest response (57.9%). In addition, statistics (19.3%) and that they learned more about grants and manuscript writing (15.8%) were identified (Table 3). Regarding what they liked least about the training, 41.2% felt there was not enough time, and 32.4% felt the training was too comprehensive.

**Table 3:** Feedback from participants’ feedback on what they liked best and liked least about the training.

Responses	No. (%)
***Liked Best (n = 57)***	
The faculty and learning structure	33 (57.9)
Statistics	11 (19.3)
Learned more about grant and manuscript writing	9 (15.8)
Study Design and fundamentals of research	2 (3.5)
Referencing	1 (1.8)
Conducive Venue	1 (1.8)
Total	57 (100)
***Liked Least (n = 34)***	
Not Enough Time	14 (41.2)
Too comprehensive	11 (32.4)
No template given for grant and proposal writing	2 (5.9)
Handouts need to be improved	2 (5.9)
No interaction between groups of participants	2 (5.9)
More topics needed on the nonacademic aspects of research	1 (2.9)
Accommodation	1 (2.9)
Statistics	1 (2.9)
Total	34 (100)

All 64 of the participants who responded to the post-training feedback said they would recommend the training to others. There was improvement in participants’ personal reflection on their skills and abilities after the training (Table 4). The participants rated their confidence in the process of conceiving and preparing research as well as writing a fundable research proposal that was mostly good and excellent after the training. They also reported that their confidence in writing a publishable scientific manuscript changed from poor to fair before the training to good and excellent after the training. One-third of the participants rated their ability to design and undertake a research project independently as good or excellent before the training. However, after the training, this ability was rated good to excellent by more than two-thirds of the participants.

**Table 4:** Participants’ self-reported confidence in their skills and abilities in various aspects of research, grant writing and publishing.

Skills and abilities	Poor (%)		Fair (%)		Good (%)		Excellent (%)	
	Before training	After training	Before training	After training	Before training	After training	Before training	After training
General skills and abilities in research	26	1	61	5	11	68	2	26
Research conception	32	0	47	0	19	47	2	53
Writing fundable research proposal	68	0	19	9	12	56	1	35
Undertake a research project independently	37	0	31	8	22	58	10	34
Writing publishable scientific manuscript	29	0	38	5	23	38	10	57

### Impact of training on research output and grant application success

**Figure 1:**
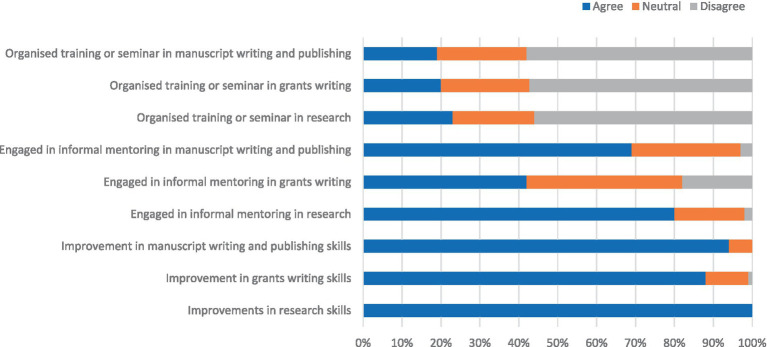
Improvements in participants’ skills and involvement in research, grant writing, and manuscript writing mentoring activities.

**Figure 2:**
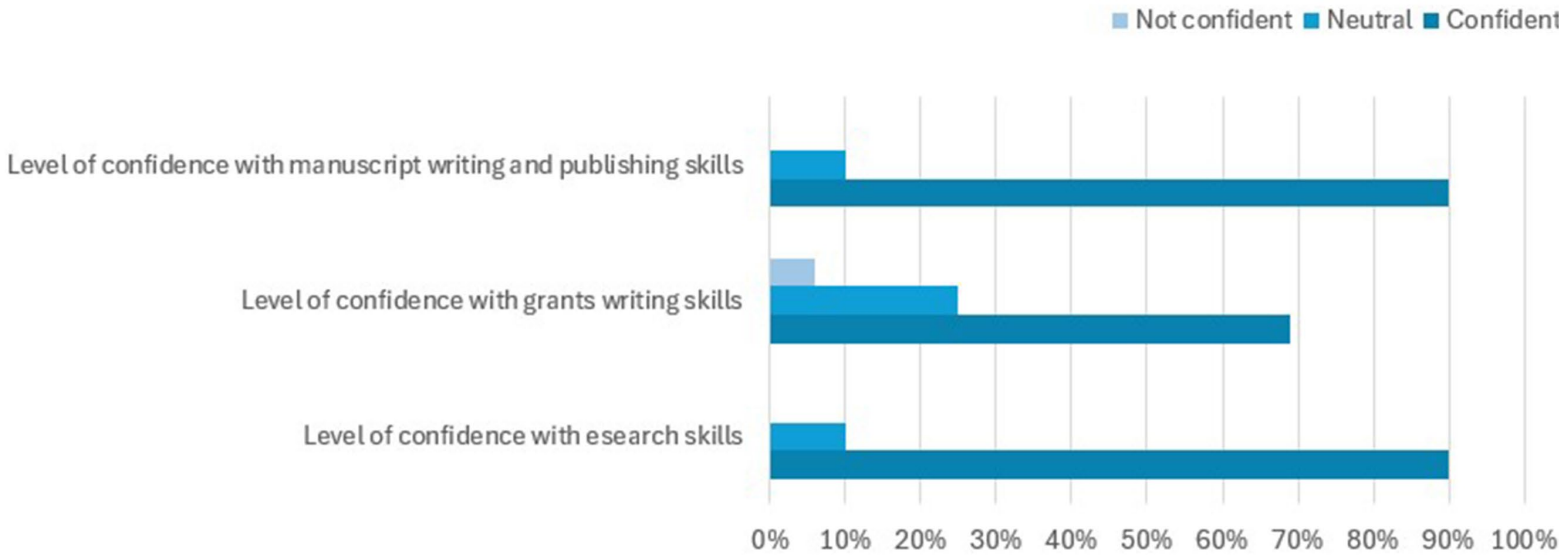
Participants’ level of confidence in their research, grant writing, and manuscript writing skills after training.

There was an 80% response to the follow-up evaluation to determine the impact of the training on research outputs and grant application success. The participants were asked to rate their level of agreement on the impact of the training on improvements in their skills in research conduct, grant writing and manuscript writing and publication. Nearly all the participants agreed that the training had improved their skills in these areas. In addition, 80% undertook informal mentoring of others in research, 69% in manuscript writing and publishing and 42% in grant writing. However, most (>50%) had not deployed any formal training or seminars in research, grant writing or manuscript writing and publishing (Figure 1). The majority of the participants felt that they were confident in research, manuscript writing and publication, and grant writing (Figure 2).

Overall, the 52 participants who completed the follow-up evaluation had 63 participant-led published manuscripts, 72 collaborative manuscripts, 14 successful participant-led grant applications and 21 successful collaborative grant applications. One hundred forty-seven research projects were designed by the participants as the lead persons (Table 5). In addition, 3 of the participants became faculty for subsequent NSOANP research training.

**Table 5:** Research outputs of 52 participants following training.

Achievements	Total No.
**Research Projects**	**254**
Research projects designed as lead persons (109, 74.1% have been commenced or completed)	147 (57.9%)
Collaborative research projects involved in (82, 76.6% have been commenced or completed)	107 (42.1%)
**Grant Applications**	**98**
Grant applications written as lead person (14, 29.2% have been successful)	48 (49%)
Collaborative grant applications involved in (21, 42% have been successful)	50 (51%)
**Manuscript Publication**	**135**
Participant led published manuscripts	63 (46.7%)
Collaborative manuscripts published	72 (53.3%)

The three most common challenges and barriers encountered by the participants were time constraints (35, 67.3%), lack of funding (19, 36.5%) and not being able to find collaborators (13, 25%) (Table 6).

**Table 6:** Challenges and barriers encountered by 52 participants in research, grant writing and publication following training.

Challenges	No. (%)
Time constraints (competing interests)	35 (67.3)
Lack of funding/lack of interested organizations in proposed research	19 (36.5)
Lack of collaborators	13 (25)
Lack of motivation	8 (15.4)
Statistics	9 (17.3)
Manuscript publishing process, e.g., Prolonged processing, rejections	6 (11.5)
Persisting difficulties in aspects of manuscript and grant writing	6 (11.5)
Ethical clearance delays	3 (5.8)
Access to subscription published literature	3 (5.8)
Lack of mentorship	3 (5.8)
Poor infrastructure for research	2 (3.8)

## Discussion

The training model used in this study was in-person, intensive and hands-on. This approach enables the mentoring of participants to work on their own research ideas and data to immediately put into practice the knowledge and skills being acquired under onsite mentoring by faculty. In addition, this in-person model ensures protected time, avoids distractions, fosters learning, and is reported to facilitate research output (14, 15). Although the COVID-19 pandemic has resulted in the increasing use of online training programmes and engagements with obvious merits, in-person training has the advantages of encouraging protected time, removing distractions considerably and avoiding technical issues associated with internet connectivity (16, 17). Such technical internet connectivity issues are common in the setting and have the potential of losing valuable learning time. However, the virtual (online) platform could be deployed to support ongoing mentoring to strengthen the knowledge and skills acquired through in-person training.

### Participants’ reception of training

The training was well received by the participants, as they particularly rated the faculty, learning structure and inclusion of statistics as the top three aspects of the training. The inclusion of grant writing was also rated as important. This is crucial, as success at grant applications has remained a challenge in LMICs, contributing to the perennial lack of research funding in the setting. Participants indicated a lack of enough time and an overly comprehensive nature of the training as what they liked least. This feedback created opportunities for improvements and restructuring for future courses.

### Impact of training

The impact of research capacity strengthening programmes has been identified to be rather low compared to that of output and outcome measures (18). However, in our training programme, the participant-reported impact was encouraging, as skills in research, writing and publishing and grant writing improved. The finding that most participants were able to engage in informal mentoring of others is desirable and encouraging and would help to cascade the benefits of the training to reach a wider pool of researchers. However, the limited deployment of formal training seminars creates an opportunity for ongoing mentoring by faculty to support participants in this regard. This would potentially further extend the impact of the training.

The participants were successful at designing both individual and collaborative research, as well as publishing research manuscripts. This is an early indication that training is gradually impacting research capacity. A lack of access to grants has been previously identified as one of the barriers to surgical research in East Africa (19). In the present report, the participant-reported success at grant applications is encouraging. This was supported by the provision of competitive small grants by a funder. The provision of such competitive small funder-administered grants allows those trained in research to quickly put into practice the skills they have acquired. This has the potential to strengthen the acquired skills and success of the applications, gradually increasing their confidence and capacity for grant writing. This model should contribute to scaling up the capacity of LMIC practitioners to compete in the wider global research funding space. Unlike other training programmes that focus on the number of trainees reached (11, 20), our approach focuses on the impact of the training on immediate and short-term rated skills, abilities, and research output. Having shown the feasibility and encouraging impact of our approach, the next steps would be to begin to significantly increase the number of those trained and encourage their involvement in mentoring others to achieve a multiplier effect.

While the achievements of this training are encouraging, ongoing mentoring and tracking of impact and progress are crucial to ensuring continuous improvements and strengthening of research capacity in the setting. In addition, developing and strengthening the research infrastructure, which is presently weak in the setting, and incorporating graduated and appropriately structured research training into the curriculum of medical schools and surgical training would be necessary to strengthen overall research capacity in the setting.

### Limitations

The main limitation of this report is that it includes a small number of participants mostly from healthcare specialties involved in cleft lip and palate care, and the findings may not be completely applicable to other healthcare specialties. However, it forms an important background to begin to build on for future training programmes.

## Conclusion

Encouragingly, the participants expressed a willingness to recommend the training to others, and there was a notable increase in their confidence levels in research and manuscript writing. This was further supported by the observed improvements in research output and grant application success. Despite these positive outcomes, barriers such as time constraints, funding limitations, and difficulties in finding research collaborators remain. Addressing these barriers presents an opportunity for strengthening training programmes and fostering continued research productivity. Going forward, such training requires ongoing support and resources to sustain the gains and overcome the persistent challenges in research capacity in a low-resource setting. Our findings emphasise already acknowledged limitations in research capacity and highlight the need to urgently invest in scaling up surgical research capacity in the setting.

## Data availability statement

The original contributions presented in the study are included in the article/Supplementary material, further inquiries can be directed to the corresponding author.

## Ethics statement

The studies involving humans were approved as part of the implementation of NSOANP by Institutional Review Board (Health Research & Ethics Committee), National Hospital, Abuja, Nigeria. The studies were conducted in accordance with the local legislation and institutional requirements. Written informed consent for participation was not required from the participants or the participants’ legal guardians/next of kin because Pre-training and post-training evaluation were integral parts of the research training programme. Participants were informed that completion of the pre- and post-training evaluations were voluntary.

## Author contributions

EA: Conceptualization, Formal analysis, Funding acquisition, Project administration, Supervision, Writing – original draft, Writing – review & editing, Resources. JS-O: Conceptualization, Formal analysis, Project administration, Writing – original draft, Writing – review & editing, Resources. NA: Conceptualization, Project administration, Writing – original draft, Writing – review & editing, Resources. AM: Writing – original draft, Writing – review & editing. MA: Writing – original draft, Writing – review & editing. OA: Writing – original draft, Writing – review & editing. NO: Funding acquisition, Writing – original draft, Writing – review & editing. IC: Writing – original draft, Writing – review & editing.
