# Supplementary material for: Strengthening surgical healthcare research capacity in sub-Saharan Africa: impact of a research training programme in Nigeria
Source: Front Med (Lausanne). 2024 Aug 9;11:1429168. doi: 10.3389/fmed.2024.1429168 (PMC11341446; doi:10.3389/fmed.2024.1429168)
Supplement: Supplementary file 1 [file Table_1.pdf]

Supplement 2: Training evaluations

Pre training evaluation

Questions

1. Age at last birthday
2. Gender: Female/Male
3. Specialty
4. Have you attended a research training before?
   Yes
   No
5. If yes to Q4 above, which year?
6. Have you attended training on grants writing before?
   Yes
   No
7. If yes to Q6 above, which year?
8. Have you attended a training on writing and publishing before?
   Yes
   No
9. If yes to Q8 above, which year?
10. Have you published any paper written by you as lead author?
    Yes
    No
11. Have you presented a paper at a conference?
    Yes
    No
12. Have you won a research grant as lead researcher before?
    Yes
    No
13. If yes, to Q12 above what is the maximum amount you have won as a grant?
14. If no to Q12, have you ever applied for a grant?
    Yes
    No
15. What are your expectations from this training? Please list
    a.
    b.
    c.
    d.
    e.
    f.
16. 16. Any other comments you may have

Post training evaluation

This survey helps us understand how future trainings may need to be adjusted to best respond to participants' needs. Please choose the most appropriate response:

1. The workshop content as a whole was:

	Strongly disagree	Disagree	Neutral	Agree	Strongly agree
Relevant					
Comprehensive					

	Strongly disagree	Disagree	Neutral	Agree	Strongly agree
Easy to understand					

Any Comments:

2. The workshop content on Fundamentals of Research was:

	Strongly Disagree	Disagree	Neutral	Agree	Strongly Agree
Relevant					
Comprehensive					
Easy to understand					

Any Comments:

3. The workshop content on Writing for Publication and Grants Writing was:

	Strongly Disagree	Disagree	Neutral	Agree	Strongly Agree
Relevant					
Comprehensive					
Easy to understand					

Any Comments:

4. The workshop handouts:

	Strongly Disagree	Disagree	Neutral	Agree	Strongly agree
Supported presentation material					
Provided useful additional information					
Were clear and well-organized					

Any Comments:

5. The workshop:

	Strongly Disagree	Disagree	Neutral	Agree	Strongly Agree
Was well paced					
Breaks were sufficient					
Was a good mix between listening and activities					
The activities were useful learning experiences					

Any Comments:

6. The facilitators (faculty) were:

	Strongly Disagree	Disagree	Neutral	Agree	Strongly Agree
Knowledgeable					
Well-prepared					
Responsive to participants' questions					

Any Comments:

7. What did you like best about this workshop, and why?

8. What did you like least about this workshop, and why?

9. Give up to three examples of how you will use the information learned during the training:

10. How satisfied were you with the accommodation and feeding?
   a) Very Satisfied
   b) Satisfied
   c) Neutral
   d) Dissatisfied
   e) Very Dissatisfied

11. Would you recommend this workshop to someone else?
   a) Yes
   b) No

Follow up evaluation

1. Age at last birthday in years:
2. Gender
   Female
   Male
3. Specialty
   a) Oral and Maxillofacial Surgery
   b) Orthodontics
   c) Paedodontics
   d) Plastic Surgery
   e) Paediatric Surgery
   f) Obstetrics & Gynaecology
   g) Ear, Nose and Throat
   h) Paediatrics
   i) Speech Therapy
   j) Nursing
   k) Nutritionist/Dietician
   l) Anaesthesia
   m) Others, Pls specify:
4. Are you from a smile train partner institution?
   Yes
   No
5. When did you attend the research training?
   a) August 2021
   b) January-February 2022
   c) August-September 2022
   d) April 2023
6. Where did you attend the training?
   a) Abuja
   b) Lagos

To what extent do you agree with the following statement:

	Statement	Strongly agree	Agree	Neutral	Disagree	Strongly disagree
7.	The research training has improved my research capability"?					
8.	The research training has improved my grant writing capacity					
9.	The research training has improved my manuscript writing and publication capability					

	Question	Very confident	Confident	Neither confident nor unconfident	Unconfident	Very unconfident
10.	What is your current level of confidence with research?					
11.	What is your current level confidence with grant writing?					

12.	What is your current level of confidence with manuscript writing and publishing?					

13. How many research projects have you designed on your own since the training?
14. How many of the above research projects have you commenced or completed?
15. How many collaborative research projects have you been involved in since the training?
16. How many collaborative research projects have been commenced or completed?
17. How many grant applications have you designed on your own since the training?
18. How many of the above (in Q17) were successful?
19. How many collaborative grant applications have you been involved in since the training?
20. How many of these (in Q19) were successful?
21. How many of these grant applications were to local funding agencies within Nigeria?
22. How many of these grant applications were to international funding agencies?
23. How many manuscripts have you led and published since the training?
24. How many published collaborative manuscripts have you been involved in since the training?

To what extent do you agree with the following statements:

	Statement	Agree	Neutral	Disagree
25.	Since the training I have informally mentored others in research			
26.	Since the training I have informally mentored others in grants writing			
27.	Since the training I have informally mentored others in manuscript writing and publishing			
28.	Since the training I have organized seminars or workshops on research			
29.	Since the training I have organized seminars or workshops on grants writing			
30.	Since the training I have organized seminars or workshops on manuscript writing and publication			

31. What 3 challenges have you faced in research, grants writing and publication since the training?
    a)
    b)
    c)
32. What additional training in research, grants writing and manuscript writing and publication do you need?
33. Provide any 3 any suggestions you feel would make the training more impactful.
    a)
    b)
    c)
