# Supplementary material for: Strengthening surgical healthcare research capacity in sub-Saharan Africa: impact of a research training programme in Nigeria
Source: Front Med (Lausanne). 2024 Aug 9;11:1429168. doi: 10.3389/fmed.2024.1429168 (PMC11341446; doi:10.3389/fmed.2024.1429168)
Supplement: Supplementary file 2 [file Table_2.pdf]

Supplement 1: Research training curriculum and schedule

DAY	TOPIC/ACTIVITY
**DAY 1**	**FUNDAMENTALS OF RESEARCH**
	The benefits of attending research trainings: personal experience
	Planning For a Research Study
	Searching and Studying/Reading the Literature
	Developing Research Questions and Hypotheses
	Sampling and Sample Sizes, Use of G Power
	Research Data Management: Data Collection, Analysis, and Interpretation
	Use of Appropriate Statistics
	Research Study Designs (Including Surveys and Controlled Trials)
	Systematic Review and Metanalysis
**DAY 2**	**FUNDAMENTALS OF GRANT WRITING & GRANTS MANAGEMENT**
	Sources of Biomedical Research Funding and Grants
	How to Develop a Fundable Research Idea
	Research Monitoring & Evaluation (Timelines, Milestones, Deliverables, Gantt Chart etc.)
	Budgeting and Grants Management
	Writing a Successful Research Grant Application
	Hands-on Work to Convert Research Ideas into Fundable Proposal
	**DATA ANALYSIS**
	Data Clean Up and Preparation for Analysis
**DAY 3**	**DATA ANALYSIS**
	Data Analysis and Statistics Software (II): SPSS, Epi Info, Stata, & Others
	Hands-on Work on Data Analysis (I): Data Clean-Up
	Hands-on Work on Data Analysis (II): Analysis Using Stata
**DAY 4**	
	Hands-on Work on Data Analysis (III): Continue
	**FUNDAMENTALS OF WRITING & PUBLISHING**
	Writing a Good Scientific Manuscript
	How to Get Your Manuscript Published: Audience, Journal, Submission, Revisions, Other Processes
	Making Effective Research Presentations at Meetings
	Ethical Issues in Research and Publishing
	Dealing with Rejection of Manuscripts & Grant Applications
**DAY 5**	
	Referencing: Formats and Software
	Writing a Good, Publishable Manuscript: Technical Tips, Dos and Don'ts
	Hands-on Work on Converting Actual Data into Publishable Manuscript
